# Exercise interveNtion outdoor proJect in the cOmmunitY for older people – the ENJOY Senior Exercise Park project translation research protocol

**DOI:** 10.1186/s12889-019-7125-2

**Published:** 2019-07-11

**Authors:** Pazit Levinger, Maya Panisset, Jeremy Dunn, Terry Haines, Briony Dow, Frances Batchelor, Stuart Biddle, Gustavo Duque, Keith D Hill

**Affiliations:** 1 National Ageing Research Institute, Royal Melbourne Hospital, Melbourne, Australia; 2 School of Primary and Allied Health Care, Monash University, Melbourne, Australia; 3 Centre for Health Policy, University of Melbourne, Melbourne, Australia; 4 Centre for Health, Informatics, and Economic Research, Institute for Resilient Regions, University of Southern Queensland, Brisbane, Australia; 5 Australian Institute for Musculoskeletal Science (AIMSS), The University of Melbourne and Western Health, Melbourne, Australia; 6 Department of Medicine-Western Health, Melbourne Medical School, The University of Melbourne, Melbourne, Australia; 7 The School of Physiotherapy and Exercise Science, Curtin University, Perth, Australia; 8 Rehabilitation, Ageing and Independent Living Centre, Monash University, Melbourne, Australia

**Keywords:** Senior Exercise Park, Physical activity, Exercise, Falls, Older people

## Abstract

**Background:**

Creating inclusive and accessible outdoor environments that provide and encourage opportunities for older adults to engage in physical activity and social interaction is important for healthy ageing. The Senior Exercise Park is outdoor exercise equipment designed specifically for use by older people that provides physical and social benefits for older people in the community, and has the potential to be used widely as a sustainable mode of physical activity. The aim of this study is to implement and evaluate the effects of sustained engagement through the use of a community-based novel outdoor physical activity program (purpose-built exercise park) for older people on physical, mental and social health and physical activity outcomes (the ENJOY project).

**Methods:**

This is a prospective pre-post design study with 12 months follow up. Adults aged ≥60 years will be recruited from the general community from the suburbs close to the Senior Exercise Parks locations in Melbourne. Participants will undergo a 12 week structured supervised physical activity program using the outdoor Senior Exercise Park equipment followed by 6 months unstructured physical activity program. Participants will be assessed at baseline, 3, 9, and 12 months. The following outcomes will be assessed: physical activity, physical function, psychosocial and mental health outcomes, falls risk and falls occurrence, participants’ feedback and satisfaction, and health care resource use.

**Discussion:**

The ENJOY trial is designed to operate in a community setting with local government engagement to maximise the usage of the exercise park and provide an outdoor space for older people to be physically active. This project will evaluate the effectiveness and sustainability of the outdoor exercise park on a range of health outcomes and its long-term usability in the community.

**Trial registration:**

This trial is prospectively registered with the Australian New Zealand Clinical Trials Registry. Trial registration number ACTRN12618001727235 registered 18th of October 2018.

**Electronic supplementary material:**

The online version of this article (10.1186/s12889-019-7125-2) contains supplementary material, which is available to authorized users.

## Background

The world’s population is ageing rapidly, with those over 65 years doubling to around 25% of the total population over the next 40 years. The number of Australians aged 65 and over is expected to increase from around 2.5 million in 2002 to 6.2 million in 2042 [1]. The beneficial effects of a physically active lifestyle (‘active ageing’) on various health outcomes are well established, with strong evidence of reduction of risk of chronic diseases, reduction of cognitive and functional decline, and improvement in mental health [2]. Although participation in regular physical activity is one of the most important health behaviours, evidence shows that older people do not regularly undertake physical activity [3] with less than 25% of older Australians meeting the recommended physical activity guidelines [4].

Creating inclusive and accessible outdoor environments that provide and encourage opportunities for older adults to engage in physical activity and social interaction is an important mechanism for promoting healthy ageing. Exercising outdoors is recommended for all ages due to its beneficial effect on mental and physical health [5]. Outdoor exercise has been shown to improve mood and self-esteem in older people [6], and community-based physical activity programs have been shown to be effective in reducing feelings of loneliness and social isolation [7]. Consequently, outdoor physical activity strategies that are novel, attractive and enjoyable for older people are needed for sustained engagement in physical activity.

A unique purpose-built outdoor exercise park program was established previously to provide a fun but physically challenging environment to support exercise in community settings, and to challenge key aspects of physical health for older people, including balance, mobility and function [8]. The Senior Exercise Park program was designed to actively promote well-being through the provision of a unique exercise mode and social support program (e.g. morning tea after exercise). This preliminary research work provided evidence in a small 18 week randomized controlled trial (RCT) that a Senior Exercise Park program can create physical and social benefits for older people in the community [9, 10]. It indicated the need for investigation of its sustained impact on physical and social health outcomes, and its potential wider usage in the community on a larger scale with local governments’ (councils) engagement. Therefore, the aim of this study is to implement and evaluate the effects of sustained engagement in physical activity on mental health and physical outcomes through the use of a novel community-based outdoor physical activity program (purpose-built exercise park) for older people (the ENJOY project). Evaluation of the exercise park usage, adherence, participants’ feedback and satisfaction, and health services cost will also be examined. Furthermore, an independent uptake and delivery of the physical activity program by local city councils and seniors’ organizations will be supported throughout the conduct of the study.

## Methods and design

All procedures involved in this trial will be conducted in compliance with National Statement on Ethical Human Resource and the Australian Code for the Responsible Conduct of Research. Ethical approval has been obtained from the Melbourne Health Human Research Ethics Committee, Melbourne (Application ID. HREC/18/MH/286, local number 2018.238). The study was designed according to the Transparent Reporting of Evaluations with Nonrandomized Designs (TREND) [11] which complements the widely adopted Consolidated Standards Of Reporting Trials (CONSORT) statement developed for randomized controlled trials [12].

### Design and setting

**Fig. 1:**
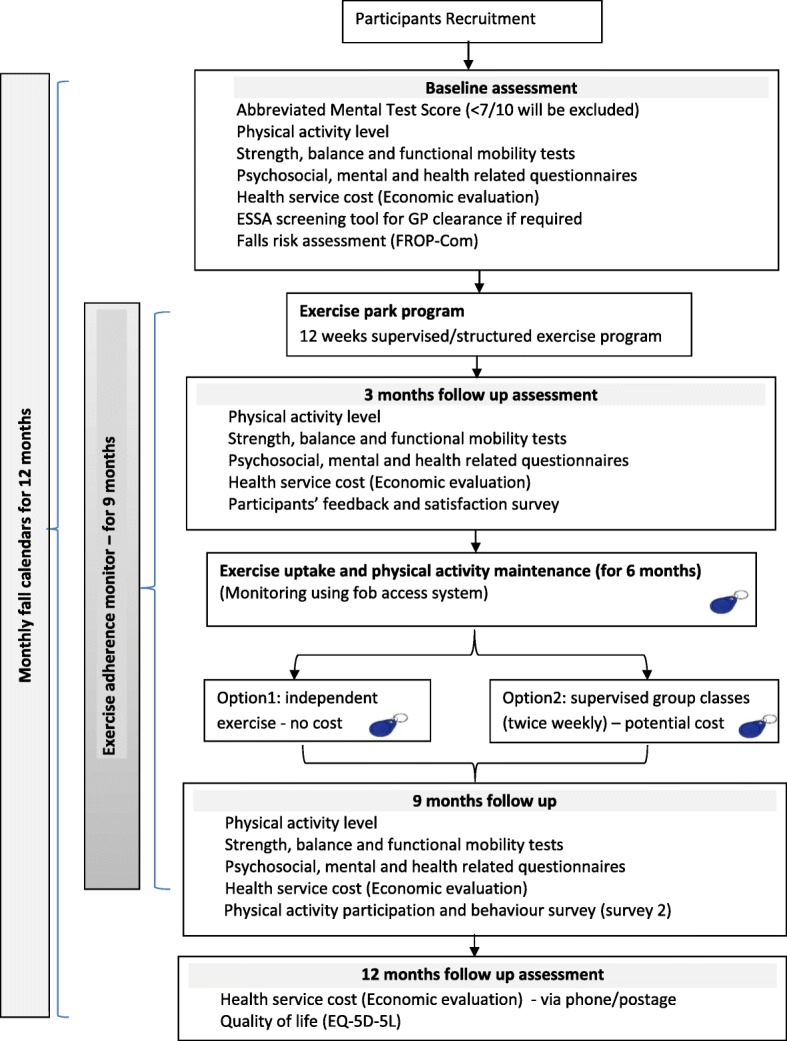
Chart flow of the ENJOY project’s design

This study is a multi-site prospective study with a pre and post intervention design with 12 months follow up. Participants will undergo a 12 week structured supervised physical activity program using outdoor exercise park equipment followed by 6 months unstructured physical activity program, including ongoing unsupervised access to the exercise park. Each exercise session will be followed by a social gathering with morning/afternoon tea. Participants will be assessed at baseline and at several follow up time points (3, 9 and 12 months) as detailed in Fig. 1.

### Study population

#### Inclusion criteria

Older people will be included in the study if they:

1. are aged 60 years and over living in the community (e.g. not living in an institution, such as a nursing home).
2. have had one or more falls in the previous 12 months or are concerned about having a fall.
3. are generally independent around the house (able to take care of themselves) and in the community (e.g. able to walk away from home to visit local stores, friends, and other local venues) and who are able to attend the outdoor exercise park.
4. do not use a walking aid (such as walking frames) or use only a single point stick used for outdoor walking;
5. do not have cognitive impairment (Abbreviated Mental Test Score > 7/10).

#### Exclusion Criteria

Older adults will be excluded from this study if they:

1. have neurological or musculoskeletal conditions limiting the person to walking less than one block;
2. have a history of stroke, Parkinson’s disease, or other neurological disorder impacting on mobility;
3. are unable to understand basic English;
4. are currently taking part in a structured resistance training and or an organised balance training program more than once a week;
5. meet the Australian physical activity recommendations of 150 min of physical activity/week [13];
6. have any documented medical condition or physical impairment that is deemed by their medical practitioner to contraindicate their inclusion.

### Recruitment

Older people will be recruited from the general community in the suburbs close to the Senior Exercise Parks location in Melbourne, Australia. Advertisements in local newspapers, council newsletters, posters displayed on notice boards, and flyers distributed to senior groups will be used for recruitment. Information will also be placed online on the councils’ and participating partners’ websites as well as associated social media platforms (e.g Facebook, Twitter). Advertisement of the research project will also be done through seniors’ group meetings and activities in the local community centres (adjacent to the Senior Exercise Park) by distribution of flyers and/or by attending sessions to provide verbal explanation of the project. Potential participants will be able to register their details if interested. Advertisements will also be placed in healthcare facilities and places with high circulation of senior citizens, and will also be mailed-out to health care practitioners in the local areas around the parks.

### Procedure

Participants who meet the inclusion criteria will attend an initial (baseline) assessment at the community centre close to their area of residence. At the baseline assessment participants will sign a consent form. Following completion of the consent form the following information will be recorded: demographic characteristics (age, gender), anthropometric measures (height and weight) previous medical history, current medication usage, socioeconomic and cultural background information (e.g. employment, level of education, country of birth, years of residency in Australia) and falls history. A flow chart of study design and study procedure and assessments are presented in Fig. 1 and Table 1 respectively.

**Table 1:**
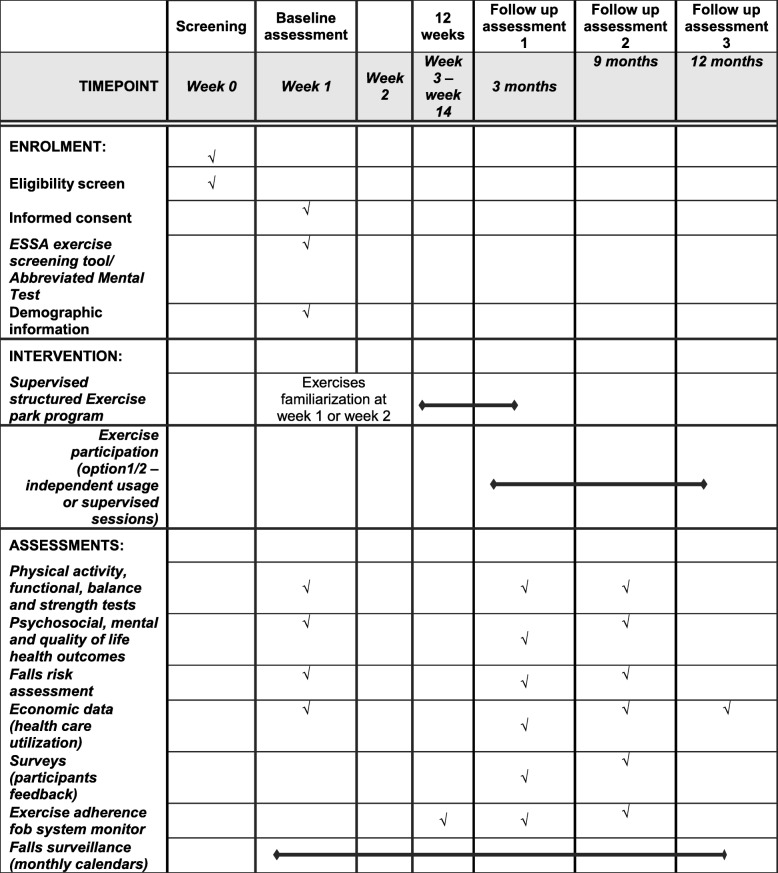
ENJOY study procedure

Abbreviated Mental Test Score (AMTS), a set of 10 questions to assess cognitive impairment (memory), will be administered on the initial assessment [14]. Participants who score < 7/10 will be excluded from the study. Participants will then be asked to complete the Exercise and Sport Science Australia (ESSA) exercise screening tool to evaluate any contraindicated medical conditions to exercise: https://www.essa.org.au/wp-content/uploads/2011/09/Screen-tool-version-v1.1.pdf. Participants answering ‘yes’ to any of the screening questions will be required to obtain medical clearance from their general practitioner prior to participation in the exercise intervention.

At baseline, 3 months and 9 months participants will undergo a comprehensive suite of physical (strength, balance, functional mobility) tests, falls risk assessment and falls history, and complete psychosocial (quality of life, enjoyment, social isolation, fear of falls, loneliness), and mental health measures (mental wellbeing, depression). In addition, they will be asked to provide feedback and complete satisfaction surveys (survey 1 and 2). Health cost/services data will also be collected (health economic evaluation). At 12 months, a final assessment will take place (via phone and or mail postage) to evaluate health cost/services as part of the health economic data collection. Monthly falls calendars will be collected from baseline for 12 months.

### Assessments

#### Primary outcome

##### Physical activity

The level of physical activity of the participants will be measured using the Community Healthy Activities Model Program for Seniors (CHAMPS) [15]. The CHAMPS is specifically designed for use in evaluating interventions that primarily aim to increase levels of physical activity in older adults. It is a reliable and valid questionnaire that is sensitive to change of the measures derived from it. The CHAMPS provides a measure of caloric expenditure (and frequency) per week in all exercise-related activities and caloric expenditure (and frequency) per week in moderate exercise -related activities.

#### Secondary outcomes

A comprehensive suite of physical function (strength, balance, functional mobility), psychosocial (quality of life, enjoyment, social isolation, fear of falls, loneliness), and mental health outcomes (mental wellbeing, depression), falls risk assessment and falls history will be assessed. In addition, physical activity participation and adherence, participant’s feedback and satisfaction, and health care resource use will also be measured, as detailed below:

##### Physical function measures

Physical measures of strength, balance and functional mobility will be assessed using the following validated tests. Rest time will be given between tests:

(i) Functional lower limb muscle strength will be assessed using the 30-s sit to stand test [16]. Participants will be asked to sit on a chair (43 cm high chair) and stand up (with arms crossed over their chest) as many times as they can for 30 s, the number of sit to stands performed during that time will be recorded.
(ii) Exercise tolerance and functional mobility will be assessed using the two-minute walk test [17]. Participants will walk in a marked area for two minutes at their comfortable pace. The distance covered during the two minutes will be recorded.
(iii) Dynamic balance will be assessed using the step test [18]. Participants will be asked to place one foot onto a 7.5-cm-high step and then back down to the floor repeatedly as fast as possible for 15 s. The number of steps completed in the 15-s period for each lower limb will be recorded.
(iv) Walking speed will be assessed using the 4 m walk test [19]. Participants will be asked to walk 4 m at their usual walking pace. Gait speed will be defined by distance (in meters) divided by time (in seconds).

##### Psychosocial, mental and quality of life health outcomes

Psychosocial, mental health and quality of life outcomes will be assessed using the following questionnaires:

(i) Health-related quality of life will be assessed using the EQ-5D-5 L [20]. The EQ-5D-5 L is a generic instrument to assess health related quality of life that comprises five dimensions (mobility, self-care, usual activities, pain/discomfort and anxiety/depression) as well as overall utility score.
(ii) *Mental wellbeing* will be assessed using the five-item World Health Organisation (WHO-5) Wellbeing questionnaire [21, 22]. The WHO-5 measures psychological wellbeing and depressive symptoms using 5 simple questions with adequate validity both as a screening tool for depression and as an outcome measure in clinical trials [23].
(iii) *Loneliness* will be assessed using the UCLA 3-Item Loneliness Scale. The UCLA Loneliness Scale includes three dimensions of loneliness: relational connectedness, social connectedness and self-perceived isolation [24, 25].
(iv) *Depression* will be assessed using the short version Geriatric Depression Scale (GDS-15) [26]. The GDS (15 point version) is a valid depression assessment tool specifically designed for older people.
(v) *Fear of falls* will be assessed using The Short Falls Efficacy Scale International (Short FES-I) questionnaire [27]. The Short FES-I is a valid and reliable 7 items scale to assess fear of falling in older people.
(vi) *Self-efficacy* barriers to exercise will be assessed using The Self-Efficacy for Exercise (SEE), a 9-item instrument that focuses on self-efficacy expectations related to the ability to continue exercising in the face of barriers to exercise [28].
(vii) *Enjoyment* will be assessed using the 8 item version Physical Activity Enjoyment Scale (PACES) [29]. The PACES is a valid instrument for assessing enjoyment in physical activity.
(viii) *Social isolation and social support* will be assessed using the short version 6 items Lubben Social Network Scale [30]. The Lubben Social Network Scale is a self-report valid and reliable measure of social engagement including family and friends.

##### Falls risk assessment and surveillance

(i) *The Falls Risk for Older People in the Community (FROP-Com)* risk assessment tool will be used to assess fall risk. The FROP-Com consists of 13 falls risk factor domains, with most risk factors scored to reflect graded risk on a 4-point scale (nil, mild, moderate, or severe) [31].
(ii) *Falls surveillance - monthly calendars -* Falls will be defined as an event when the participant ‘inadvertently comes to rest on the ground, floor or other lower level’ (WHO Global Report on Falls Prevention in Older Age [32]). Participants will be given calendars to record any falls experienced each day on a monthly falls calendar, which will be returned to the investigators via postage-paid mail each month. If a fall is recorded, or a calendar is not returned within two to three weeks of the end of any month, a research staff member will administer a standardised questionnaire via telephone to collect or clarify details of the circumstances of any falls.

##### Participants’ feedback and satisfaction surveys (3 months and 9 months)

Survey 1

At the completion of the 12 week exercise intervention participants will be asked to fill an evaluation form (survey) that collects feedback about the exercise program (duration, frequency, difficulty of the exercises), usability of the exercise park (in terms of location, safety), facilities/amenities available (water, benches, toilet etc), and any suggestions for further improvement of the site. The survey will include 25 questions using a 5-point Likert scale as well as 7 open-ended questions for additional comments/suggestions.

Survey 2

At 9 months following the baseline assessment, participants will be asked to complete another evaluation form (survey 2) that gathers information about their exercise habits (if they continued using the exercise park, exercise frequency and duration). The survey will include 8 questions using a 5-point Likert scale, 7 multiple-choice questions as well as 5 open-ended questions for additional comments/suggestions.

##### Health care resource use and productivity

Health care costs will be measured using Medicare and Pharmaceutical Benefits Scheme database extractions for publicly subsidized primary health services. Hospitalisations will be measured using participant self-report of diagnosis and days spent in hospital. Productivity costs will be measured using the iMTA Productivity Cost Questionnaire [33] which includes three modules measuring productivity losses of paid work due to 1) absenteeism and 2) presenteeism and 3) productivity losses related to unpaid work. Participants will be asked to complete this questionnaire at baseline and 12 month follow up assessments. Home nursing, allied health and community service use will be captured using self-report. For the 12 month assessment, the form will be posted to the participants in addition to the EQ-5D-5 L. A reply paid envelope will be enclosed so participants can post these directly to the research team.

### Exercise Park intervention

#### The senior Exercise Park

**Fig. 2:**
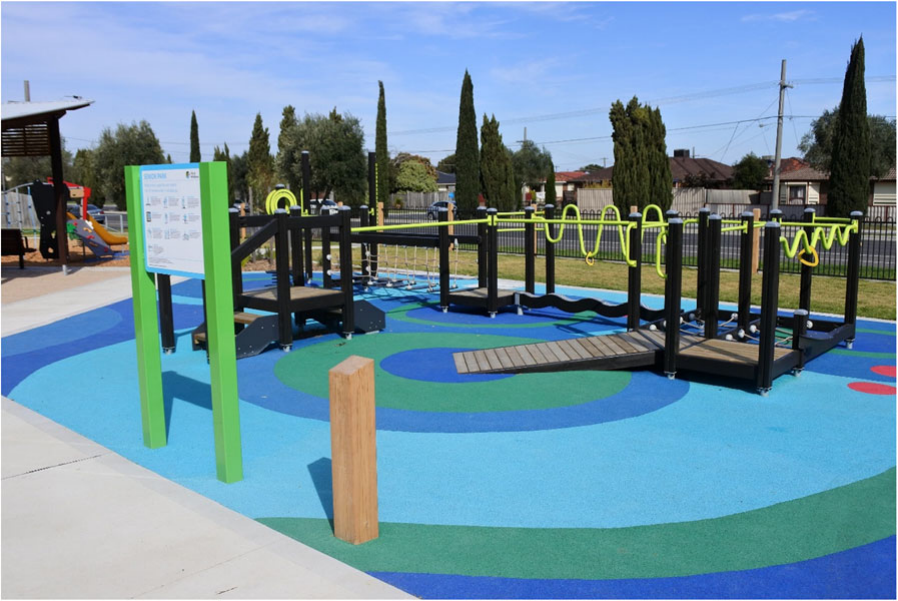
The Senior Exercise Park (Lark Industries and Lappset Group) at Thomastown, Melbourne Victoria

The Senior Exercise Park equipment (Lark Industries, Australia) is outdoor playground equipment specifically designed for older people to improve strength, balance, joint movements and overall mobility and function (Fig. 2). It comprises multiple equipment stations that target specific function or movement (upper and lower limb) such as shoulder range of movement, static and dynamic balance (unstable surfaces), functional movements of walking up/down stairs, and sit to stand. The Senior Exercise Park complies with the Australian Standard for playground equipment AS4685 and has undergone and passed a safety assessment and testing under Australian regulations. The floor surfaces are non-slippery rubber (softfall) suitable to any playground equipment (Fig. 2). The softfall is designed to absorb impact from falls and protect against injury in a playground environment. It consists of a dual layer structure, the wear layer which is the visible top surface and the underneath layer is the shock absorbing layer which is made from a recycled rubber.

The equipment is available outdoors and is safe to use by any age group. Our previous feasibility study found the exercise park to be safe for use by older people (aged 60 years and over and with increased risk of falls) with no adverse events [10]. The exercise park equipment has been installed in two public locations and a third location in a retirement living and aged care facility respectively: Barry Rd. Community Centre, Thomastown, Melbourne (under the municipality of Whittlesea City Council); Central Park Community Centre, Hoppers Crossing Melbourne (under the municipality of Wyndham City council); and Leith Park, St Helena Melbourne (Old Colonists’ Association of Victoria).

#### 12 weeks structured supervised exercise program

Participants will undergo a 12-week supervised exercise intervention program twice a week using the Senior Exercise Park. The exercise program will be delivered by a qualified exercise instructor (accredited exercise physiologist or physiotherapist). Participants will perform exercises that focus on strength, balance, coordination, mobility and flexibility similar to our previous work [8]. The exercise park sessions will be provided twice a week (each class approximately 1 to 1.5 h duration). Each session will consist of 5–10 min warm-up exercises, followed by 45–75 min on the equipment stations, and will conclude with 5–10 min of cool down exercises. The exercise classes will include 6–10 participants and will be circuit-based. A familiarisation session will be organised for each participant prior to commencement of the exercise program. The initial level of the exercise difficulty will be determined during the familiarisation session and will be tailored to the capabilities of the participant. To maximise social interaction and enjoyment, morning/afternoon tea will be organised following the exercise sessions. Participants will be encouraged to use the exercise park and exercise as often as they like outside the structured sessions. Participants will be provided with an exercise program-recording sheet that will contain instructions with associated illustrations as well as space to fill in dates and other details.

##### Familiarisation and exercise intensity

A familiarisation session will be organised for each participant prior to commencement of the exercise program. The exercises will follow the guidelines of the Australian Position Statement of exercise for falls prevention [34]. Participants will be introduced to the 10-point Borg Rating of Perceived Effort (RPE) scale [35] at their familiarisation session. The initial level of the exercise difficulty will be tailored to the capabilities of the participant with the primary consideration of safety. Adjustment of the exercises (i.e. increase in intensity and difficulty) will be made based on the participant individual progression. RPE will be used to determine the intensity of each exercise where participants will be encouraged to exercise with a RPE between 4 and 7/10.

##### Individual and group exercise progression

Each exercise station will include two exercises and will be performed twice by each participant. Examples of the stations and the exercises can be found https://youtu.be/PaYuCMtnlYk. Two participants will be allocated to each station such that each participant will perform one exercise for the allotted time and then swap over, repeating each exercise twice before rotating to the next station. In circumstances where there are an uneven number of participants, one participant will be assigned to a station. Participants will be given a resting period of up to 60 s between exercise stations, which will be adjusted according to program progression. The duration of each exercise will also increase based on program progression. New exercises will be gradually introduced to the participants every 1–2 weeks. Details of the exercises (stations and description), exercise duration, rest and progression are provided in Tables 2, 3, 4, Additional file 1. Although similar, there are some minor variations in the structure of the exercise park set-up in each of the three sites, which means that exercise programs may not be exactly the same across the three sites.

**Table 2:** Stations and paired exercises

Station Number	Exercise 1	Exercise 2
1	Pull-ups	Hand roll
2	Balance stool	Calf raises + Finger steps
3	Gangway	Core twister
4	Snake pipe (big wave)	Balance beam
5	Shoulder arches	Snake pipe (small wave)
6	Ramp + Net + Climb	Step up
7	Push ups	Sit to stand
8	Stairs	Hip extension
9	Taps on platform	Hip abduction

**Table 3:** Duration of exercise time and rest time during the 12 weeks structured exercise program

Week Number	Session number	Set/exercise Time	Rest Time
1 to 2	1–4	60 s	60 s to change over and rest
3 to 6	5–12	60 s	≤30 s change over and rest
7 to 9	13–18	75 s	≤30 s change over and rest
10 to 12	19–24	90 s	≤30 s change over and rest

**Table 4:** Increase in the number of stations during the 12 weeks structured exercise program

Week Number	Session number	Exercise stations
1 and 2	1–4	Stations 1 to 6
3 and 4	5–8	Stations 1 to 7
5 and 6	9–12	Stations 1 to 8
7–12	13–24	Stations 1 to 9

#### Exercise uptake and physical activity maintenance

Exercise participation after completion of the structured supervised program – 3 months to 9 months

After completion of the 12 week program, participants are expected to be familiar with the equipment, the exercises and their physical abilities and therefore able to exercise independently and safely if they choose to do so. At the completion of the structured 12 weeks exercise program participants will be given two options to choose from to continue their physical activity.

Option 1 – independent unsupervised access and usage of the exercise park in participants’ own preferred time, free of charge.

Option 2: access to twice a week supervised exercise sessions on the exercise park with a potential cost of $5–8 per session. The inclusion of cost will be dependent on the participating council and will be equivalent to the price that older adults would have to pay to attend a group exercise program that is publicly subsidized through community health services in Australia. Hence, this option is to simulate what the likely cost and participation for an ongoing group program in real life. During this 6 month follow-up period, participants will be informed of weekly times when the exercise instructors will be available at the Senior Exercise Park. Participants can then attend on their preferred time/sessions. At these sessions, the exercise instructors will supervise participants and provide advice regarding exercise progression to fit individual’s progression needs.

#### Participation rate (adherence) and exercise monitor

##### During the 12 weeks supervised exercise program

Frequency of physical activity will be determined from daily attendance logs kept by the exercise instructor. Overall adherence to the structured exercise program will be defined by the number of sessions attended: where 100% adherence is if participant attended 24 sessions.

##### Monitoring exercise uptake following the 12-week exercise program for 6 months – fob access system

Adherence and exercise uptake for 6 months post intervention will be monitored using a fob access system (CityWatch Security, Victoria Australia). The fob access system will include a scanner/card reader (Asperio RF card reader) installed at each site (mounted on a bollard), a control panel (Integriti Control Panel) within a secure cabinet installed at a location (external wall) nearby to the card reader (receives signals from the RF card to the control panel), and specialized software (Integriti Professional Software) installed in the head office (National Ageing Research Institute researchers’ office). Participants will then be assigned their individual identification key (fob) which they will be able to tap at the card reader each time they access the Senior Exercise Park. Their access will then be recorded and monitored (thereby electronically monitoring access).

### Safety considerations and adverse events

#### Weather elements

In extreme weather conditions (e.g. heavy rain, extreme heat (above 30 °C)) if deemed by the exercise instructor as unsafe to exercise, sessions will be cancelled. Our pilot study results in Melbourne indicated that weather did not affect overall adherence (80%) for the exercise sessions with less than 10% of sessions cancelled [10]. In summer in Melbourne, classes will be conducted in the morning and late afternoon (to avoid the high temperatures around the middle of the day), and shade-cloth cover and/or other sun-smart behaviours will be facilitated. Participants will be encouraged to bring their own sunscreen and water to sessions (although these will also be available from the exercise instructor at supervised sessions). In circumstances where sessions will be cancelled, or during a holiday period, makeup sessions will be organised towards the end of the program (up to two weeks or 4 sessions).

#### Adverse events

##### Muscle soreness

Following the exercise sessions, short duration muscle soreness will be expected and is a typical response of the body to exercise in people who have done little or no exercise, or people who are performing an unfamiliar type of exercise. Instances of severe muscle soreness reported by the participant will be recorded.

##### Falls

Any falls during the delivery of the structured supervised exercise programs and during the independent usage phase of the Senior Exercise Park will be recorded.

##### Cardiorespiratory adverse reaction

Any report of difficulty breathing that does not settle quickly with rest, new or unrelenting chest pain, or acute changes in the level of consciousness during the session, may precede a serious medical emergency such as cardiac arrest or stroke. In this event the session will be stopped immediately and an emergency response will be initiated. A potentially serious event will be defined if the participant reported difficulty breathing but symptoms settled quickly with rest and their clinical signs (respiration rate, heart rate, oxygen saturation) remain normal. The participant will be able to elect to complete the session provided these symptoms settled quickly with rest and clinical signs remain normal. A serious adverse event will be defined if symptoms have not settled and medical emergency care was required. All adverse events will be recorded.

### Sample size calculation

The power calculation is based on increasing physical activity level (CHAMPS) by the end of the 9 month follow-up period. A previous study using the CHAMPS reported mean differences of 687 (SD difference 1509) and 487 (SD difference 1196) energy (calories) expenditure per week in all activities and in moderate intensity activities respectively following 6 months physical activity program for older people [15, 36]. To account for any potential variation between the ENJOY exercise park program and the latter reported community physical activities as well as potential variation in the sample population (people from different multicultural backgrounds) [37] a reduction of 20–25% (500 and 400 cal/week in all activities and in moderate intensity activities respectively) was applied to these outcomes. Using this conservative approach, an effect size d = 0.33, 90% power and alpha =0.05,a sample size of 98 participants is required (G*Power, two tail). To account for potential 15% drop out, a sample of 113 will be recruited. As such, we aim to recruit a sample of 37–38 participants in each participating site.

### Statistical analysis

For the primary outcome of overall physical activity score and the physical, mental, and health outcome measures, regression analyses with data clustered within individual participants will be used to determine if there are differences between scores collected at baseline assessment and at 9 month follow-up. Moreover, repeated measures analysis of variance will be used to examine the effect of the exercise program on physical activity level, physical, mental and psychosocial and health outcomes between the other time points (baseline, 3 months, 12 months). Information collected about park usage, participant’s feedback and exercise adherence will be reported using descriptive statistics (frequency of usage, % of adherence). The outcome variables will be assessed for normality prior to analysis and transformed accordingly. Data will be analysed using SPSS version 25.0 (IBM Corp, NY, USA). Multiple imputation will be used to account for missing data at the 9 month follow-up assessment.

#### Economic analysis

The economic evaluation will take the form of an incremental cost-utility analysis taken from the societal perspective over a 6 month pre intervention vs 6 month post intervention time-horizon. This analysis will estimate the cost per quality adjusted life year gained from providing the Senior Exercise Park intervention and supervised exercise program (incorporating the optional continuation of the supervised program). Change in quality adjusted life year will be modelled using data from EQ-5D-5 L utility instrument scores collected at baseline, and 12 month assessments. Hospitalisations measured using participant self-report of diagnosis and days spent in hospital will be converted to a cost using the National Weighted Activity Unit funding approach (https://www.ihpa.gov.au/what-we-do/national-weighted-activity-unit-nwau-calculators). Productivity in paid and unpaid labour will be calculated using responses to the iMTA questionnaire [33]. Change in community service use, home nursing, allied health will be valued using market rates for comparable services provided through the private sector.

## Discussion

An holistic approach is required to support uptake of physical activity to create sustained changes in behaviour for older people. Fun and enjoyment of social interaction are key motivators for older people to take part in physical activity [38] and they prefer to exercise with their same age group [39]. The Senior Exercise Park is an innovative outdoor exercise equipment that has been previously shown in a small 18 week RCT with short follow-up, to be effective in improving strength and function in older people as well as being a socially enjoyable mode of physical activity [9, 10]. This innovative approach requires local governments to be actively involved in the design of outdoor space that is specifically suitable for older age. However further research is needed to evaluate the effectiveness and sustainability of the outdoor Senior Exercise Park on a range of health outcomes and its long term usability in the community. Local Councils and community organisations are the most likely organisations to install Seniors Exercise Parks, and it is important that they have evidence to inform decision making regarding wide reach implementation of such innovative physical activity within their communities.

There are substantial methodological challenges that often inhibit implementation of physical activity programs into practice, these include: lack of evidence of transferability of trial results to community setting, insufficient local expertise to roll out community exercise programs, and inadequate infrastructure to integrate evidence based programs into community practice [40]. As such interventions that are designed to be conducted in the community setting with community engagement can represent a ‘real world’ design that can potentially sustain participation beyond the trial period. In fact, community based interventions have shown to be effective in increasing and promoting physical activity [41, 42]. Therefore, to maximise the usage of the Senior Exercise Park and increase community engagement, the ENJOY trial is designed to run in a community setting. Outcomes from this study can shape the future of age friendly outdoor space in the Australian communities.

## Additional file

Additional file 1: Exercise descriptions and progression. (PDF 662 kb)

## Data Availability

Not Applicable.

## Abbreviations

AMTS: Abbreviated Mental Test Score
CHAMPS: Community Healthy Activities Model Program for Seniors
CONSORT: Consolidated Standards Of Reporting Trials
ENJOY: Exercise interveNtion outdoor proJect in the cOmmunitY for older people
EQ-5D-5 L: The 5-level EQ-5D
ESSA: Exercise and Sport Science Australia
FROP-Com: The Falls Risk for Older People in the Community
GDS: Geriatric Depression Scale
PACES: Physical Activity Enjoyment Scale
RCT: randomized controlled trial
RPE: Rating of Perceived Effort
SD: standard deviation
SEE: Self-Efficacy for Exercise
Short FES-I: Short Falls Efficacy Scale International
TREND: Transparent Reporting of Evaluations with Nonrandomized Designs
UCLA: University of California at Los Angeles Loneliness Scale
WHO: World Health Organization
