# Supplementary material for: Exercise interveNtion outdoor proJect in the cOmmunitY for older people – the ENJOY Senior Exercise Park project translation research protocol
Source: BMC Public Health. 2019 Jul 11;19:933. doi: 10.1186/s12889-019-7125-2 (PMC6624877; doi:10.1186/s12889-019-7125-2)
Supplement: Supplementary file 1 — Exercise descriptions and progression. (PDF 662 kb) [file 12889_2019_7125_MOESM1_ESM.pdf]

Additional file 1: Exercise descriptions and progression

| Stations/Exercise | Description | Functional relevance | Progressions |
|---|---|---|---|
| **Pull Ups** 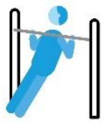 | Participant places hands (overgrip) on the bar approximately shoulder width apart and toes on the line closest to the bar. Participant pulls body up towards the bar and then lowers with control | Strengthens arms, back and core muscles | 1. Increase distance of feet from the bar<br>2. Feet placed on 10cm high step<br>3. Single leg pull ups |
| **Hand Roll** 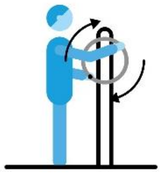 | Participant places hands on both grips. Begin by turning each handle simultaneously forward/ backward | Improves mobility of the ribcage, shoulders and elbow joints | 1. Turn handles simultaneously in forward motion<br>2. Turn handles simultaneously in backward direction<br>3. Turn handles 180 degrees out of phase (pedalling)- Forward<br>4. Turn handles 180 degrees out of phase (pedalling)- Backward<br>5. Turn handles in opposite directions – one forward and one back<br>6. Repeat steps 1-4 with tandem stance<br>7. Repeat steps 1-4 standing on one leg only<br>8. Repeat steps 1-4 standing on one foot with heel raised Stand facing the centre of one ring (on the side) and use one hand only in single leg stance (+/- heel rise) |
| **Balance Stool** 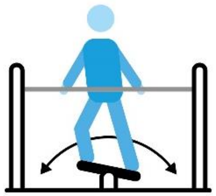 | Participant balances with both feet on the unstable stool while rocking side to side with 2 hands on the bar | Improves balance, strengthens the hip and deep muscles that support the spine | 1. Reduce hand support (2 hands > one hand > no hands)<br>2. Hands overhead<br>3. Alternating hands over head<br>4. Repeating with rocking movements in different directions (Rotations > Forward/Backward)<br>7. Lifting foot up (one at a time) while rocking from side to side<br>8. Single leg (repeat steps 1-4 progressions)<br>9. Mini squats (repeat steps 1-4 progressions) |

| Stations/Exercise | Description | Functional relevance | Progressions |
|---|---|---|---|
| **Calf Raises and Finger Steps** 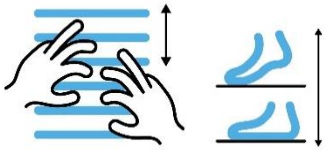 | Participant raises up onto toes and at the same time climbs the finger steps to reach the highest point possible | Improves finger agility and shoulder mobility while improving stability and balance | 1. Facing the bar, double leg calf raise, 2 hands<br>2. Facing the bar, tandem stance and raise, 2 hands<br>3. Facing the bar, single leg calf raise, 2 hands<br>4. Side on to the bar, double leg calf raise, 1 hand<br>5. Side on to the bar, single leg calf raise, 1 hand |
| **Gangway** 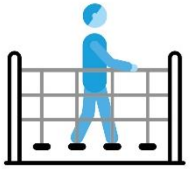 | Participant walks along the rickety bridge surface | Unstable surfaces, improves dynamic balance | 1. Reduce hand support (2 hands > one hand > no hands)<br>2. Lift knee up between steps<br>3. Partial lunge between steps |
| **Core Twister** 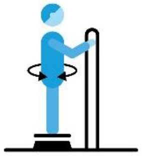 | Twist the body from side to side | Improves spinal mobility and core strength | 1.Two hand support<br>2. One hand support, hand reaching out and back, alternate sides<br>3. No Hands<br>4. Single leg, repeat steps 1-3<br>5. |
| **Snake Pipe – Big Wave** 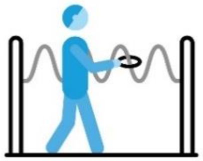 | Participant moves the ring from one end to the other without touching the bar | Strengthens and mobilises the shoulders, challenges balance | 1. Side facing, walking, and looking forward<br>2. Side facing, walking heel to toe<br>3. Side facing, walking on toes, looking forward<br>4. Side facing, walking backward<br>5. Side facing with head turns, repeat steps 1-4 |

| Stations/Exercise | Description | Functional relevance | Progressions |
|---|---|---|---|
| **Balance Beam** 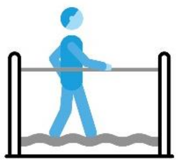 | Participant walks back and forth along the beam. | Improves balance while navigating (or walking on) uneven surfaces | 1. Hand for support, normal walking<br>2. Heel to toe walking<br>3. Reduce hand support (2 hands > one hand > no hands)<br>4. Walking on toes (hand support if needed)<br>5. Walking on toes and knees up |
| **Shoulder Arch** 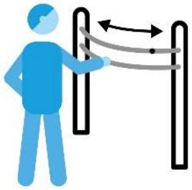 | Participant moves the ball along the bar. | Improves shoulder flexibility | 1. Facing convex side of the bar (outside), hold each ball with one hand, move the balls as far out as possible and then back into the middle and cross over. Alternate hands on top and bottom<br>2. Hold each ball with one hand, move both balls to right/left<br>3. Side facing, move the ball as far forward as possible, then move the ball backward as far as possible while rotating your trunk. Alternate sides.<br>4. As above, in single leg stance (leg closes to the bar), alternate<br>5. As above, in single leg stance (leg furthest to the bar), alternate |
| **Snake Pipe – Small Wave** 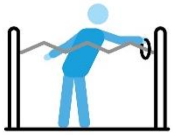 | Participant faces the bar with feet apart and moves the ring from one end to the other without touching the bar, bending the front knee to reach further. | Strengthens and mobilises the shoulders and back, improves reaching skill and balance | <u>Facing the snake pipe:</u><br>1. Feet apart, change hands in the middle<br>2. Feet apart, same hand reaching across the body<br><u>Side on to the snake pipe:</u><br>3. Feet together, reaching forward, alternate sides<br>4. Feet together, reaching forward and backward, alternate sides<br>5. Standing on one leg (innermost), reaching forward backward<br>6. Standing on one leg (outermost), reaching forward backward |

| Stations/Exercise | Description | Functional relevance | Progressions |
|---|---|---|---|
| **Ramp + Net + Climb**<br>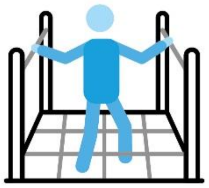 | Participant walks up the ramp and steps down either through the net or on to the ropes | Improves dynamic balance, obstacle negotiation and foot clearance | <u>Walking through the net without hitting the ropes:</u><br>1-3. Narrow stance, ranging from two hand support, one hand support and no hand support<br>4-6. Wide stance, ranging from two hand support, one hand support and no hand support<br><u>Walking balancing on the ropes:</u><br>7-9. Narrow stance, ranging from two hand support, one hand support and no hand support<br>10-12. Wide stance, ranging from 2 hand support, one hand support and no hand support |
| **Step Up***<br>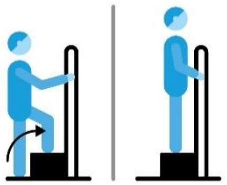 | Participant steps up and down the platform | Strengthens the legs, improves functional mobility for stairs and getting in and out the bath/bus, raises the heart rate | 1. Alternating legs, with hand support<br>2. Alternating legs, no hand support<br>3. 5 on each leg (and alternate), no hand support<br>4. 10 on each leg (and alternate), no hand support<br>5. Increase steps on each side by 5<br>*6. Add ankle weight or increase step height |
| **Push Ups**<br>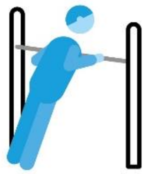 | Place hands on the bar approximately shoulder width apart and toes on the line closest to the bar. Control chest down towards the bar and then push away. | Strengthens arms, back and core muscles | 1. Increase feet distance from the bar<br>2. Standing on 10cm high step<br>3. Single leg push ups |
| **Sit to Stand**<br>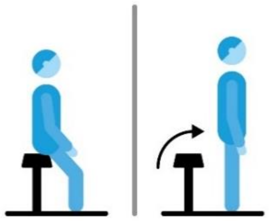 | Participant sits and stands up from the seat or stands to squat and touch the bench. | Strengthens the muscles of the hips, knees and back, improves ability to stand from a seated position | 1. Sit to stand (STS) with hand support<br>2. STS with arms in front of the body<br>3. STS with arms crossed on the chest<br>4. Place feet on a 10cm high block (steps 1-3 hand progressions)<br>5. Squatting to touch the bench (steps 1-3 hands progressions)<br>6. Increase height of block (will vary depends on client's height) |

| Stations/Exercise | Description | Functional relevance | Progressions |
|---|---|---|---|
| **Stairs *** 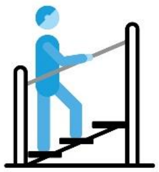 | Participant steps up and down the stairs | Helps with daily activities, raises the heart rate and strengthens the muscles of the legs | 1-3. Stepping up and down slowly ranging from two hand support, one hand support and no hand support<br>4-6. Stepping up every second step ranging from two hand support, one hand support and no hand support<br>*7. add ankle weight |
| **Hip Extension*** 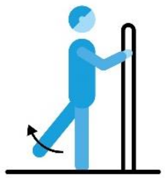 | Participant, with control and keeping back and knee straight and foot flexed, slowly take leg backwards tightening bottom muscles. | Strengthens the hip extensor muscles | 1. Extending leg backward, alternating legs, 10 on each<br>2. Hold (3 sec) at the top of the movement, alternate legs, 10 on each<br>3. Hold (3 sec) at the top of the movement, alternate legs, 15 on each.<br>4. Repeat levels 1-3 with added Thera band<br>*increase Thera band resistance (red/green/blue) |
| **Taps on Platform** 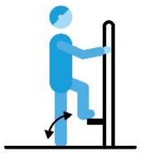 | Participant stands with feet together in front of the platform. Tap lightly on the platform one foot at a time while using hand support | Improves foot clearance, balance and ability to use stairs and to get in and out the bath/bus | 1. Tap on platform, alternating legs, using hand support.<br>2. Tap on platform, alternating legs, arms in front of the body.<br>3. Tap on platform, alternating legs, arms above head.<br>4. Increase speed of movement (safely)<br>5. Tap on a higher platform |
| **Hip Abduction*** 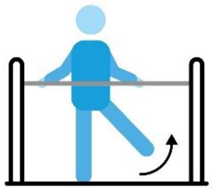 | Participant moves their leg side to side with straight knee | Strengthens the hip abductor muscles | 1. Abducting the leg to the side, alternating legs, 10 on each (slow speed)<br>2. Hold at the top of the movement, alternating legs, 10 on each<br>3. Hold at the top of the movement, Alternating legs, 15 on each<br>4. Repeat levels 1-3 with added Thera band<br>*increase Thera band resistance (red/green/blue) |
